# Untargeted Contrast-Enhanced Ultrasound *Versus* Contrast-Enhanced Computed Tomography: A Differential Diagnostic Performance (DDP) Study for Kidney Lesions

**DOI:** 10.6061/clinics/2020/e1489

**Published:** 2020-02-18

**Authors:** Li Jin, Feng Xie

**Affiliations:** I Department of Ultrasound, the First People’s Hospital of Tian Shui GanSu Province, Tianshui, 741000, China; II Department of Ultrasound, the Second People’s Hospital of Lanzhou, Lanzhou, 730000, China

**Keywords:** Chromophobe Renal Cell Carcinoma, Contrast-Enhanced Computed Tomography, Contrast-Enhanced Ultrasound, Histopathology, Papillary Renal Cell Carcinoma, Renal Cell Carcinoma

## Abstract

**OBJECTIVES:**

Histopathology is the ‘gold standard’ for diagnosing renal cell carcinoma but is limited by sample size. Contrast-enhanced ultrasound can differentiate malignant and benign lesions, but the Chinese guidelines on the management of renal cell carcinoma do not include this method. The purpose of this study was to compare the diagnostic parameters of contrast-enhanced ultrasound against those of contrast-enhanced computed tomography for detecting kidney lesions, with histopathology considered the reference standard.

**METHODS:**

Patients with suspected kidney lesions from prior grayscale ultrasonography and computed tomography were included in the analysis (n=191). The contrast-enhanced ultrasound, contrast-enhanced computed tomography, and histopathology data were collected and analyzed. A solid, enhanced mass was considered a malignant lesion, and an unenhanced mass or cyst was considered a benign lesion. The Bosniak criteria were used to characterize the lesions.

**RESULTS:**

Contrast-enhanced ultrasound and contrast-enhanced computed tomography both detected that 151 patients had malignant tumors and 40 patients had benign tumors. No significant differences in the tumors and their subtypes were reported between contrast-enhanced ultrasound and histopathology (*p*=0.804). Chromophobe renal cell carcinoma was detected through contrast-enhanced computed tomography (n=1), but no such finding was reported by contrast-enhanced ultrasound. A total of 35 cases of papillary renal cell carcinoma were reported through contrast-enhanced ultrasound while 32 were reported through histopathology.

**CONCLUSIONS:**

Contrast-enhanced ultrasound might be safe and as accurate as histopathology in diagnosing kidney lesions, especially renal cell carcinoma. Additionally, this study provides additional information over histopathology and has an excellent safety profile.

**Level of evidence:**

III.

## INTRODUCTION

Lesions are characterized by their location in the body, size, and cause. Lesions can occur anywhere in the body, but lesions usually develop in the soft tissues i.e. the brain, skin, kidney, and lung. Renal lesions can be benign or malignant. To differentiate these lesions, various techniques are used, e.g., histopathology, ultrasound, computed tomography (CT) scans, and magnetic resonance imaging (MRI). The features that distinguish benign lesion from malignant lesions are their size and growth pattern (1).

**Figure 1:**
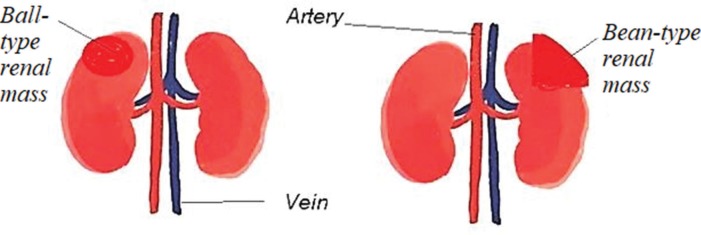
Pictorial presentation of renal lesions. Ball-type and bean-type renal lesions.

There are two distinctive growth patterns of kidney lesions, ball and bean. The most common renal solid masses are ball-shaped, usually renal cell carcinoma (RCC), which deform the bean-shaped contour part of the kidney. The bean-type growth pattern retains the renal shape of the kidney and infiltrates into the renal parenchyma (Figure 1). Ball-type lesions can be easily detected, but it is difficult to detect bean-type renal masses using conventionally available techniques (2). A large tumor size is considered a hallmark feature of malignancy. It is of utmost importance to characterize the size of the tumor using imaging method(s) and/or histopathology (3).

**Figure 2:**
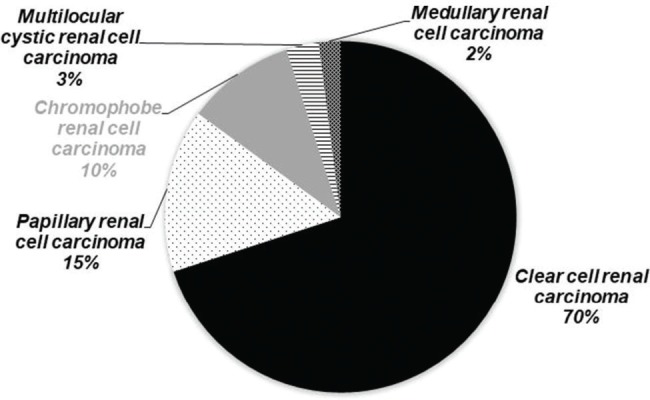
Frequency of occurrence of different types of renal cell carcinoma in the population.

RCC is most common kidney lesion and is associated with the age of the patients. Usually, RCC is reported in adults aged 60 years or older. There are various subtypes of RCC classified by the World Health Organization (WHO). The most commonly observed RCC is clear cell carcinoma, and 70% of RCC cases are clear cell carcinoma. This carcinoma is heterogeneous and extensive. Chromophobe (5% of the cases) and papillary RCC (10-15% of the cases) are also quite common. Smaller papillary RCC is hypovascular and homogenous. Smaller papillary RCC usually presents as microcysts. In contrast, chromophobe RCC manifests as sharp lesions with lobes. The least frequently observed types are renal medullary RCC and multilocular cystic RCC (Figure 2) (4).

The most frequently observed renal lesion that is not RCC is angiomyolipoma. It contains fat and smooth muscles. The second most commonly observed benign renal lesion that is not RCC is oncocytoma. It is essential to differentiate and identify various types of renal lesions to provide proper treatment to the patient (2).

Ultrasound waves are sound waves with frequencies above 20,000 Hz. The application of contrast medium to conventional ultrasound is a common technique used in medical science. For sonographic examinations, the contrast agents generally used are microbubbles with a size of 10-12 µm. These microbubbles are made of saline, perfluorocarbons, or nitrogen and are supported with the help of polymer shells, lipids, or proteins. Gas-filled contrast media are commercially available and are administered intravenously. High contrast can be observed with the use of these agents due to the differential reflection of ultrasound waves from interfaces existing between different substances (5).

Contrast-enhanced ultrasound (CEUS) is an advanced technique and has various advantages over conventional techniques like traditional unenhanced ultrasound, MRI, or histopathology. CEUS is able to differentiate between renal solid masses, renal lesions, small cysts, complex cysts, and pseudolesions. This is why CEUS has become a potential tool for observing and characterizing renal lesions (6).

CEUS is a relatively advanced and is an emerging technology. It is able to depict the kidney physiology and anatomy. Contrast agents were introduced in 1996 for ultrasonography and are still being used, and new agents are emerging. CEUS causes no nephrotoxicity or radiation hazards and does not cause discomfort to the patient (7).

Histopathology is considered as the ‘gold standard’ in various clinical diagnostic processes in research laboratories. It is used as a benchmark to confirm results obtained through various advanced processes. However, histopathology is a laborious task that can take anywhere from hours to days and certainly depends on the expertise of the histopathologists. This is why sometimes artifacts due to toned tissue sections or inconsistent staining appear. Histopathology is also limited by the sample size that can be analyzed, and only a small area of interest can be observed. On the other hand, there are techniques like CEUS that allow observation of the whole organ (8).

CEUS has the ability to differentiate between benign and malignant tumors and can determine the degree of malignancy, especially for focal liver lesions, as suggested by various studies (9). The guidelines and recommendations for the use of CEUS in clinical practices have put great emphasis on extrahepatic applications (10). Diverse applications of CEUS in modern medicine have been discussed in this document. No single radiologist is able to master all of these applications, but individually, these evaluations have the potential to replace CT and MRI (11). The guidelines also recommend the use of CEUS in the diagnosis of kidney lesions and observation of the pancreas and urinary tract (12).

CEUS can serve as a useful tool for the differentiation between pseudotumors and neoplasms. For example, CEUS can distinguish between dromedary hump and column of Bertin in the kidney (extensions of the renal cortical tissue that are involved in separating nephrons). These features are normal in 50% of the population (13). However, dromedary hump can also mimic renal neoplasms. These structures are the budges on the lateral border of the kidney and are benign lesions (14).

If there is a similarity between the enhancement pattern and adjacent renal parenchyma, then the mass under study is a pseudotumor and not a neoplasm. CEUS can detect lesions that are not detectable by grayscale ultrasound (9). The distinction between malignant renal tumors and benign lesions is necessary because of the high prevalence of kidney cancer in the Chinese population (15). If the lesion is identified in an earlier stage, then treatment is possible. Therefore, it is necessary to differentiate between malignant and benign lesions, and CEUS can accomplish this task. Benign renal lesions are very common, and 30% of the reported lesions were benign (16).

The objectives of this retrospective study were to evaluate the efficiency of CEUS in differentiating between benign and malignant tumors, identifying the lesion subtypes, and determining the malignant nature and to assess the accuracy and sensitivity of CEUS in comparison to those of contrast-enhanced computed tomography (CECT), considering histopathology as the ‘reference standard’.

## MATERIALS AND METHODS

### Ethics approval and consent to participate

The designed study protocol (SPL/CL/17/19 dated 21 May 2019) was approved by the Second People’s Hospital of Lanzhou Review Board. Informed consent forms were signed by the enrolled patients for pathology, radiology, anesthesia, and publication of the study in all formats of the publication house, including the publication of personal data and images during hospitalization, irrespective of time and language. The study reporting adhered to the laws of China, the Strengthening the Reporting of Observational Studies in Epidemiology (STROBE): Cross-sectional Studies statement, and the v2008 Helsinki Declaration.

### Inclusive criteria

Patients aged above 18 years with elevated serum creatinine who had undergone prior imaging (grayscale ultrasonography and CT) as a part of routine clinical care to evaluate kidney structure and size and had suspected kidney lesions were included in the analysis.

### Exclusion criteria

Individuals with active cardiac disease (unstable angina, myocardial infarction, and severe arrhythmia), lung cancer, respiratory distress syndrome, emphysema, hypersensitivity response against contrast agents, especially Levovist^®^ (a mixture of galactose microparticles, air bubbles, and palmitic acid) and iohexol, were excluded from radiology. Patients who had a history of drug abuse were also excluded from radiology. Pregnant women and breastfeeding females were also excluded from radiology. Individuals in critical condition or intensive care unit status and those who declined to undergo radiological and/or pathological examinations were excluded.

### Data collection

Patients who gave consent for diagnostic procedures during hospitalization filled out a consent form and provided general information about their lifestyle. The grayscale ultrasonography, CT, CEUS, CECT, and histopathology data were collected.

### Untargeted contrast-enhanced ultrasound

The patients were instructed to lay down on a bed, and a 2.5 g bolus Levovist^®^ (Schering AG, Berlin, Germany) was administered intravenously. When the microbubbles passed through the imaging system, the signal oscillated and reflected, and the ultrasound images were recorded by a Sonoline Elegra medical ultrasound system (K981528, Siemens Healthcare, Hamburg, Germany) using 4 MHz abdominal transducers (Siemens Healthcare, Hamburg, Germany). The arterial phase ultrasound image was recorded at a 2.8 MHz frequency, 100% maximum output with a single point focus, and a 5/s frame rate, which was decreased to 2/s and eventually to 1/s. The echogenicity was converted into a high-contrast image of the area of interest (17). Sonographers (minimum 5 years of experience) acquired the scans.

### Contrast-enhanced computed tomography

The patients were instructed to lay down on a bed, 100 mL iohexol (755 mg/mL, Omnipaque™, GE Healthcare, Chicago, IL, USA) was administered intravenously at 4 mL/s, and the images were recorded by a 16-slice CT scanner (GE Healthcare, Chicago, IL, USA) (18). Radiologists (minimum 5 years of experience) acquired the scans.

### Image analysis

Grayscale ultrasound, CT, CEUS, and CECT images were uploaded on a RadiAnt DICOM Viewer-Chinese system (Medixant Maciej Frankiewicz, Poznan, Poland), and the Bosniak criteria were used to characterize the lesions (18). A solid, enhanced mass was considered a malignant lesion, and an unenhanced mass or cyst was considered a benign lesion (19). Sonographers and radiologists (minimum of 5 years of experience) were involved in the image analysis.

### Histopathology

With a 20G, 20 cm semiautomatic biopsy gun (Supercore, Varay Laborix, Bourges, France), the tissues were collected by physicians (minimum 5 years of experience). After dissection, the tissues were placed in a fixative buffer (Carnoy’s solution, Mark Specialist, Berlin, Germany) to prevent decay. The part of the tissue that was expected to bring about accurate manifestations of the disease was removed (2-3 µm) and placed on the slide using formalin (Mark Specialist, Berlin, Germany) as a fixative. Moisture was removed from the samples using varying concentrations of ethanol (Mark Specialist, Berlin, Germany) and xylene (Mark Specialist, Berlin, Germany). Wax (Mark Specialist, Berlin, Germany) was introduced at the end to adhere the tissue sample on the slide. The slide was stained with hematoxylin (Mark Specialist, Berlin, Germany) and eosin (Mark Specialist, Berlin, Germany) to enable microscopic observation. After observation, digital cameras captured advanced images of the histopathological specimen (2).

The histopathology of the renal lesions was characterized on the basis of different patterns of staining and observable appearances of the slides (20) (Table 1). Pathologists (minimum 5 years of experience) were involved in the histopathological analysis.

### Statistical analysis

The correlation of the CEUS, CECT, and histological results was analyzed based on the expert evaluations of the type and subtype of lesions. The chi-square independence test (2) was performed for constant data. The results were considered significant at the 95% confidence level.

## RESULTS

### Enrollment

**Figure 3:**
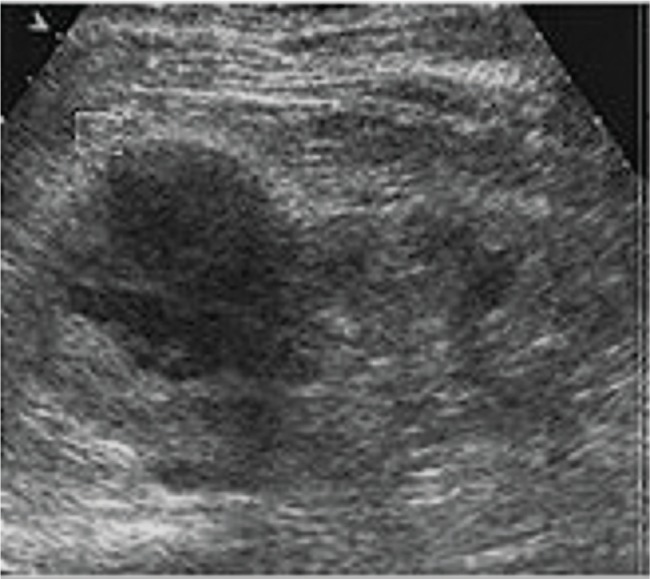
Grayscale ultrasonography showing a suspected mass-mimicking lesion in the right kidney in a 53-year-old man with clear cell renal carcinoma.

**Figure 4:**
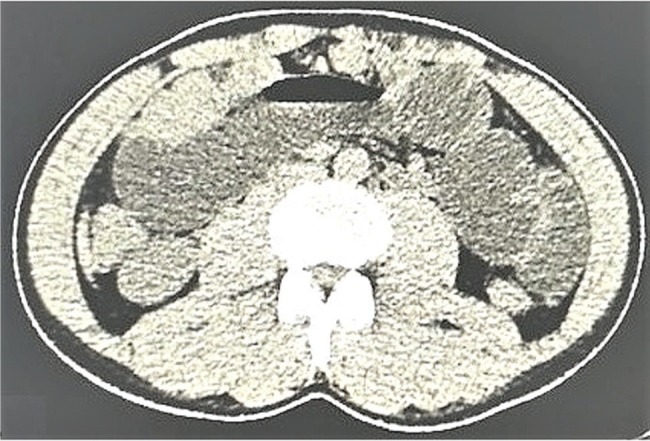
Computed tomography showing a suspected mass-mimicking lesion in the right kidney in a 55-year-old man with clear cell renal carcinoma.

**Figure 5:**
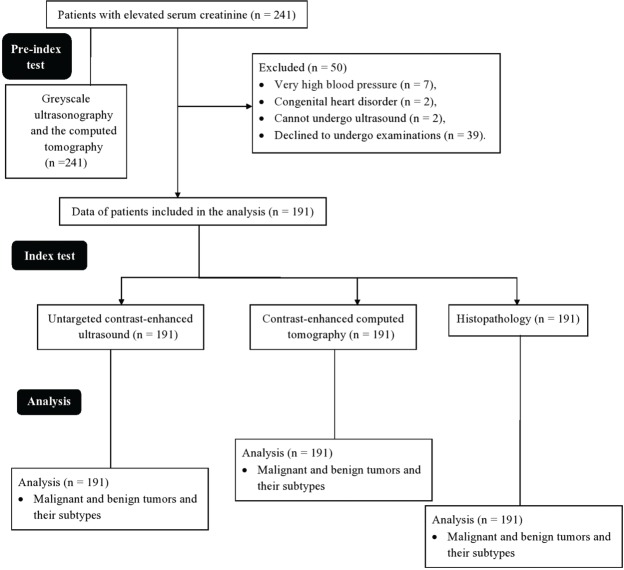
Flow diagram of the study.

From 15 January 2017 to 31 December 2018, patients enrolled at the Second People’s Hospital of Lanzhou, Lanzhou, China and the First People’s Hospital of Tian Shui GanSu Province, Tianshui, China underwent imaging examinations as a part of routine clinical care to evaluate kidney structure and size. Grayscale ultrasound (Figure 3) and CT (Figure 4) images suggested kidney lesions in 241 patients with elevated serum creatinine levels during routine clinical care. Of these patients, seven were excluded due to very high blood pressure, two were excluded because of congenital heart disorders, two women were breastfeeding and could not undergo CEUS/CECT, and 39 patients declined to undergo radiological examinations. The grayscale ultrasound, CT, CECU, CECT, and histopathology data of 191 patients were included in the analysis. A flow diagram of the study is presented in Figure 5.

### Demographic and clinical characteristics

Out of 191 patients, 137 (72%) were men, and 54 (28%) were women. The mean age of the enrolled patients was 63.51±12.25 years, 120 patients were smokers (15 cigars per day), and 71 had never smoked any kind of tobacco product. The other demographic and clinical characteristics of the enrolled patients are reported in Table 2.

### Untargeted contrast-enhanced ultrasound

**Figure 6:**
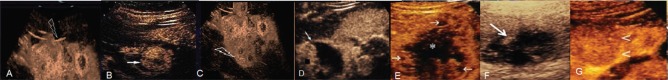
Contrast-enhanced ultrasound images of the kidney. A: Clear cell renal carcinoma. B: Papillary renal cell carcinoma-type I. C: Papillary renal cell carcinoma-type II. D: Infiltrative urothelial renal cell carcinoma. E: Collecting duct renal cell carcinoma. F: Angiomyolipoma. G: Oncocytoma.

CEUS showed tumor blood flow in all of the patients. CEUS detected 151 patients with malignant tumors and 40 patients with benign tumors. A total of 104 individuals had clear cell carcinoma, 35 had papillary RCC (papillary RCC-type I and papillary RCC-type II), six individuals had collecting duct carcinoma, six patients had infiltrative urothelial carcinoma, 27 individuals had angiomyolipoma, and 13 patients were diagnosed with oncocytomas, according to CEUS (Figure 6).

### Contrast-enhanced computed tomography

**Figure 7:**
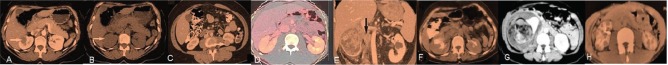
Contrast-enhanced computed tomography of the kidney. A: Clear cell renal carcinoma. B: Papillary renal cell carcinoma-type I. C: Papillary renal cell carcinoma-type II. D: Chromophobe renal cell carcinoma. E: Collecting duct renal cell carcinoma. F: Infiltrative urothelial renal cell carcinoma, G: Angiomyolipoma. H: Oncocytoma.

Contrast-enhanced computed tomography detected 151 patients with malignant tumors and 40 patients with benign tumors. A total of 104 individuals had clear cell RCC, 35 patients had papillary RCC (papillary RCC-type I and papillary RCC-type II), six individuals had collecting duct carcinoma, six had infiltrative urothelial carcinoma, 27 individuals had angiomyolipoma, 12 were diagnosed with oncocytomas, and one individual had chromophobe RCC (Figure 7).

### Histopathology

**Figure 8:**
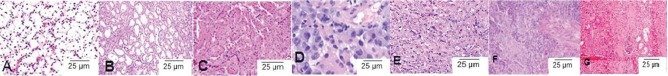
Histopathological examinations. A: Clear cell renal carcinoma (lipid-rich cytoplasm with sharply visible nuclei), B: Papillary renal cell carcinoma-type I (spindle-shaped basophilic cells with scarce cytoplasm), C: Papillary renal cell carcinoma-type II (spindle-shaped organized cells covered with granular eosinophils with distinct nuclei), D: Chromophobe renal cell carcinoma (large cells with pale surroundings and perinuclear halos), E: Collecting duct renal cell carcinoma (irregular channels were lined by high-grade cuboidal to hobnail cells with eosinophilic cytoplasm). F: Infiltrative urothelial renal cell carcinoma (sessile, polypoid, fungating, ulcerated, and infiltrative lesion with the neoplastic cells separated by a desmoplastic stroma). G: Oncocytoma (nested architecture with a central scar and bland cytology).

The results of CEUS and CECT for renal lesions had some contradictions, and these contradictions were clarified using histopathology. The histopathology results showed that out of 191 patients, 148 had malignant lesions, and 43 had benign lesions. Forty-three individuals had histopathology results indicative of benign tumors and suggestive of angiomyolipoma (n=30) and oncocytomas (n=12) (Figure 8).

### Diagnostic parameters

No significant differences between CEUS and histopathology (*p* = 0.804) or between CECT and histopathology (*p*=0.804) were reported for tumors and their subtypes. Histopathology suggested that the results of CEUS were more accurate for diagnosing kidney lesions. CEUS had a sensitivity of 0.99, and CECT had a sensitivity of 1 compared to histopathology. The specificities of the diagnostic methods are presented in Table 3.

## DISCUSSION

CECT detected one patient with chromophobe RCC and 12 patients with oncocytoma, but CEUS detected oncocytoma in 13 patients. The results of histopathology revealed that the CECT results were correct; chromophobe RCC was found in one patient, and oncocytoma was found in 12 patients. These results were consistent with the results of a previous prospective study (21). Chromophobe RCC is a sharp lesion with lobes, resembles oncocytoma, and has contrast enhancement that is similar to that of oncocytomas (4), which was why sonographers considered oncocytoma for a RCC and observed oncocytomas in 13 patient images, while histopathologists had observed only 12 oncocytoma slides. This is due to the incorrect interpretation of the CEUS expert.

When diagnosing clear cell RCC, CEUS, CECT, and histopathology (n=104) had equal efficiency and accuracy. These results were consistent with the results of previous prospective studies (21,22) and retrospective studies (23). This might be because clear cell RCC is the most common and well-studied type of kidney lesion.

**Figure 9:**
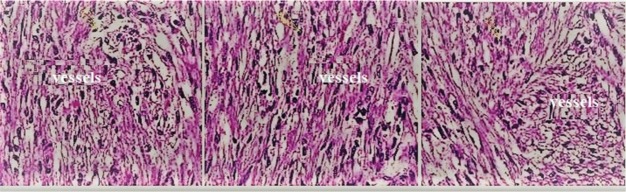
Histopathological examinations of the three contradictory cases of angiomyolipoma that were actually of papillary renal cell carcinoma. All three cases consisted of varying amounts of smooth muscle cells, mature fat and thick-walled vessels. Additionally, abnormal vessels were often hyalinized and thick with eccentric lumen, and smooth muscle cells appeared to originate and “radiate” off the vessels. The yellow arrow indicates smooth muscle cells.

CEUS found 35 cases of papillary RCC cases, while histopathology found 32 (and 3 as angiomyolipoma (Figure 9)). CECT was also performed for contradictory angiomyolipoma samples (n=3) because there was an unclear increase in attenuation between unenhanced and enhanced CT (24), which suggested that the three contradictory samples were actually papillary RCC. Pathologists diagnosed these three cases as benign tumors with an angiomyolipoma subtype because all three cases consisted of varying amounts of smooth muscle cells, mature fat and thick-walled vessels. Additionally, abnormal vessels were often hyalinized and thick with eccentric lumen, and smooth muscle cells appeared to originate and “radiate” off the vessels. The reduced efficiency of various histopathology steps, i.e., toned tissue, inconsistent staining, and incorrect interpretation, may lead to misdiagnosis. In precursor lesions, pathological changes have not yet fully developed, and histological assessments are subject to diagnostic uncertainty (8). CEUS may be more efficient in determining the subtype of the malignant tumor to assist in administering the correct treatment/surgery for RCC, and it is important to be aware of the limitations of histopathological examinations in the diagnosis of RCC.

However, for collecting duct RCC and infiltrative urothelial RCC, the results were self-explanatory and consistent for both CEUS and CECT examinations. The histology results were also similar and suggested that CEUS was equally sensitive, accurate and efficient as the CECT method in detecting RCC, but the Chinese guidelines on the management of RCC (15) and USFDA (the United States of Food and Drug Administration) (18) do not include CEUS for the diagnosis of RCC; however, CEUS is more accurate in differentiating between benign and malignant tumors (6). The study suggested that CEUS has the potential to replace CECT for the diagnosis of RCC.

A total of 27 cases of angiomyolipoma were reported via CEUS and CECT each. In contrast, histopathology detected 30 cases. Twelve cases of oncocytomas were reported via histopathology and CECT each, but CEUS reported 13 cases. These results were consistent with the results of other prospective studies (21,22). One case of chromophobe RCC was detected through histopathology and CECT each, but CEUS failed to detect chromophobe RCC. The differentiation of non-RCC from RCC is problematic for CEUS (25). CEUS and CECT have substantially different probabilities of detecting non-RCC (26). Further research is required for the diagnosis of non-RCC.

The study reported that CEUS had a higher sensitivity but lower specificity than CECT. The results of the study were in line with the results of a pilot study (18). This abnormal parenchymal enhancement on CEUS is required to overcome issue of specificity.

No adverse effects occurred within the scope of the study. Levovist^®^ has no nephrotoxicity issues because it is secreted out after the destruction of the microbubbles. Levovist^®^ does not make use of iodine; thus, it does not affect or disrupt the function of the thyroid gland. Levovist^®^ is foreign to the body, and the immune system can initiate a hypersensitivity reaction (10). Hypersensitivity reactions were reported in 0.002% of the cases in previous abdominal studies (27). The incidence of hypersensitivity reactions observed with Levovist^®^ is much lower than that observed with iohexol (28). Patients with elevated serum creatinine were included in this study and underwent CECT with iohexol. The safety of patients during the diagnosis of renal lesions with iodinated contrast agents must be considered.

There were several limitations in this study that need to be reported, including the retrospective nature, lack of prospective analysis and chance of bias. The comorbidities also affect the development of kidney lesions, but this study did not perform comorbidity analyses. Intra- and interobserver agreement is required for ultrasound examinations. Levovist^®^ has a shorter half-life than iohexol and is destroyed during scanning, making continuous scanning impossible. Second generation contrast agents (e.g., SonoVue, Optison, Sonazoid, and Definity) include some inert gases other than air and have made continuous scanning possible (29).

## CONCLUSIONS

Contrast-enhanced ultrasound possesses no nephrotoxicity and may not affect thyroid gland function. The use of first-generation contrast agents, i.e., Levovist^®^, in this study was safe and made continuous scanning possible for a short interval of time. This study has helped reach the conclusion that contrast-enhanced ultrasound might be safe and as accurate as histopathology. Additionally, these results depict a very powerful tool with an excellent safety profile that provides additional information to contrast-enhanced computed tomography and histopathology for diagnosing kidney lesions, especially renal cell carcinoma.

## AUTHOR CONTRIBUTIONS

All authors read and approved the manuscript for publication. LI was the project administrator and contributed to the conceptualization, software usage, formal analysis, and literature review of the study. FX contributed to the validation, resource management, data curation, investigation, and literature review of the study and drafted, reviewed, and edited the manuscript for intellectual content. Both authors agree to be accountable for all aspects of work and ensure integrity and accuracy.

## Figures and Tables

**Table 1:** Interpretation of the histopathological examinations.

Observations	Interpretation
Lipid-rich cytoplasm with sharply visible nuclei	Clear cell renal cell carcinoma
Spindle-shaped basophilic cells with scarce cytoplasm	Papillary renal cell carcinoma type I
Spindle-shaped organized cells covered with granular eosinophils with distinct visible nuclei	Papillary renal cell carcinoma type II
Irregular channels lined by high grade cuboidal to hobnail cells with eosinophilic cytoplasm	Collecting duct renal cell carcinoma
Sessile, polypoid, fungating, ulcerated, and infiltrative lesion with the neoplastic cells separated by a desmoplastic stroma	Infiltrative urothelial renal cell carcinoma
Hemorrhagic cysts with a line of epithelial cells and clear cytoplasm	Medullary cystic renal cell carcinoma
Varying amounts of smooth muscle cells (appear to originate and “radiate” off the vessels), mature fat and thick-walled abnormal vessels	Angiomyolipoma
Nested architecture with a central scar and bland cytology	Oncocytomas
Large cells with pale surroundings and perinuclear halos	Chromophobe renal cell carcinoma

**Table 2:** Anthropological, demographic, and clinical characteristics of the enrolled patients.

Characteristics		Value
Data of the patients included in the study		191
Age (years)	Minimum	50
	Maximum	69
	Mean ± SD	63.51±12.25
Sex	Male	137 (72)
	Female	54 (28)
Ethnicity	Han Chinese	173 (91)
	Mongolian	16 (8)
	Tibetan	2 (1)
Habit	Smokers	120 (63)
	Never smoked any kind of tobacco product	71 (37)
*Serum creatinine (mg/dL)	Male	1.9±0.05
	Female	1.8±0.06
Body mass index (kg/m^2)^		25.12±1.89
Comorbidities	Diabetes	25 (13)
	Hypertension	65 (34)
	Hyperlipidemia	76 (40)
	Cardiovascular disease	22 (12)

Constant parameters are presented as frequencies (percentages), and continuous parameters are presented as the mean ± SD.

* Normal value: 0.6-1.1 mg/dL in females and 0.7-1.3 mg/dL in males.

**Table 3:** Tumors and their subtypes examined by the diagnostic methods.

Types of tumor		Histopathology	Imaging modalities			
			Untargeted contrast-enhanced ultrasound		Contrast-enhanced computed tomography	
Data of the patients included in the study		191	191	* *p-value*	191	* *p-value*
Total lesions						
Malignant lesions		148 (77)	151 (79)	0.804	151 (77)	0.804
Benign lesions		43 (23)	40 (21)		40 (23)	
Renal cell carcinoma						
Clear cell renal carcinoma		104 (54)	104 (54)	0.991	104 (54)	0.991
Papillary renal cell carcinoma	Type-I	12 (7)	15 (9)		15 (9)	
	Type-II	20 (10)	20 (10)		20 (10)	
Collecting duct renal cell carcinoma		6 (3)	6 (3)		6 (3)	
Infiltrative urothelial renal cell carcinoma		6 (3)	6 (3)		6 (3)	
Non-renal cell carcinoma						
Angiomyolipoma		30 (16)	27 (14)	0.58	27 (14)	0.976
Oncocytoma		12 (6)	13 (7)		12 (6)	
Chromophobe renal cell carcinoma		1 (1)	0 (0)		1 (1)	

Parameters are presented as frequencies (percentages).

The chi-square independence test was used for statistical analysis.

A *p* value <0.05 was considered significant.

* With respect to histopathological data.

The Bosniak criteria were used to characterize lesions.

Ultrasonographers, radiologists, and pathologists (all had a minimum of 5 years of experience) were involved in analyzing the diagnosis parameters.
